# Safety and efficacy of tislelizumab plus chemotherapy for first‐line treatment of advanced esophageal squamous cell carcinoma and gastric/gastroesophageal junction adenocarcinoma

**DOI:** 10.1111/1759-7714.13690

**Published:** 2020-10-12

**Authors:** Hai‐Bo Qiu

**Affiliations:** ^1^ Department of Gastric Surgery Sun Yat‐sen University Cancer Center; State Key Laboratory of Oncology in South China; Collaborative Innovation Center for Cancer Medicine Guangzhou China

Upper gastrointestinal tract (UGI) cancers, including esophageal squamous cell carcinoma (ESCC) and gastric/gastroesophageal junction (G/GEJ) adenocarcinoma, are the top seven most common cancers in the world.^1^ The incidence of ESCC and gastric cancer (GC) varies widely worldwide and the highest incidence is seen in China.^2^ Both of these UGI cancers have a very poor prognosis, mainly because the early symptoms of these diseases are vague and nonspecific.^3^, ^4^ Various treatment guidelines recommend combination chemotherapy as the first‐line treatment for advanced ESCC and GC.^5^, ^6^ Patients with metastatic/recurrent ESCC usually receive combination chemotherapy of cisplatin plus paclitaxel or 5‐fluorouracil.^7^ The chemotherapy regimen containing fluoropyrimidine and platinum is the standard regimen for patients with locally advanced/metastatic GC.^8^ However, the prognosis for advanced ESCC or GC patients who receive standard treatment is still unsatisfactory.

Growing evidence from cancer immunology research shows that immune checkpoint proteins, especially programmed cell death protein 1 (PD‐1) and programmed death‐ligand 1 (PD‐L1), play a crucial role in antitumor immunotherapy. PD‐L1/PD‐1 inhibitors can induce durable antitumor responses across multiple types of cancer, including advanced ESCC and GC.^9^, ^10^ Moreover, combining chemotherapy with PD‐L1/PD‐1 inhibitors can synergistically improve antitumor activity.^11^ Currently, two ongoing clinical trials are investigating pembrolizumab plus chemotherapy as the first‐line treatment for advanced G/GEJ adenocarcinoma.

Tislelizumab is a novel engineered anti‐PD‐1 monoclonal antibody. Compared with other PD‐1 antibodies, it has the weakest binding ability to FcγR on macrophages, thus avoiding antibody‐dependent phagocytosis and thereby greatly reducing the possibility of anti‐PD‐1 therapy resistance.^12^ Two phase I/II clinical trials (NCT02407990^13^ and CTR20160872^14^) demonstrated that tislelizumab (200 mg administered intravenously every three weeks) was well tolerated with few adverse events with a promising antitumor effect in both Asian and non‐Asian patients with ESCC or GC. In a study recently published in *Clinical Cancer Research*, entitled “Tislelizumab Plus Chemotherapy as First‐line Treatment for Advanced ESCC and G/GEJ Adenocarcinoma”,^15^ NCT03469557 team led by Professor Jianming Xu from the Chinese PLA General Hospital in China conducted a phase II, multicohort clinical trial to assess the safety and preliminary antitumor activity of tislelizumab plus 5‐fluorouracil and cisplatin in patients with advanced ESCC or plus oxaliplatin and capecitabine in patients with advanced G/GEJ adenocarcinoma.

In this study, a total of 30 patients were enrolled between 18 July 2017 and 22 March 2018. Among them, 15 (50%) patients suffered ESCC and 15 (50%) suffered G/GEJ adenocarcinoma. As of 31 March 2019, there were 11 (73.3%) ESCC patients and 11 (73.3%) G/GEJ adenocarcinoma patients in whom treatment was discontinued because of disease progression (54.5%), treatment emergent adverse events (27.3%), withdrawal of consent (13.6%), and noncompliance (4.5%). The most common adverse events associated with tislelizumab and/or chemotherapy drugs were anemia (*n* = 18), followed by decreased appetite (*n* = 17), nausea (*n* = 16), and asthenia (*n* = 15). Moreover, the objective response rates and disease control rates for both ESCC and G/GEJ adenocarcinoma were 46.7% and 80.0%, respectively. The median duration of response for ESCC patients was 12.8 months (95% confidence interval [CI] = 3.5–12.8 months). However, this study did not report the duration of response for G/GEJ adenocarcinoma patients as this data was not yet mature.

This study demonstrated that the adverse events caused by tislelizumab plus chemotherapy in the first‐line treatment of advanced ESCC and G/GEJ adenocarcinoma were controllable. The reported adverse events related to the combination therapy were consistent with the known adverse events associated with chemotherapy alone. Therefore, no adverse events related to tislelizumab alone were observed in this study. All patients with reported adverse events had only mild‐to‐moderate symptoms, except for one ESCC patient who had hepatitis B virus (HBV) infection at the same time, which led to the occurrence of fatal hepatic dysfunction related to the investigator's treatment. Additionally, with the exception of one case death from pneumonia related to chemotherapy, all adverse events had resolved after study treatment discontinuation.

Previous studies show that first‐line chemotherapy for advanced ESCC (cisplatin or oxaliplatin in combination with paclitaxel or 5‐fluorouracil) and advanced GC (a platinum agent in combination with a fluoropyrimidine) have objective response rates of 37.0%–58.0% and 25.0%–75.0%, respectively. However, the median duration of response is only 4.0–7.0 months and 9–13 months, respectively.^7^, ^13^ The objective response rate of tislelizumab plus chemotherapy in the present study was equivalent to that of traditional first‐line chemotherapy (46.7% vs. 37.0%–58.0% or 25.0%–75.0%), whereas the median duration of response was longer than traditional first‐line chemotherapy (12.8 months vs. 4.0–7.0 months or 9–13 months). Although we cannot directly compare these data due to the potential differences between patient populations, these data still show that tislelizumab combined with chemotherapy can contribute to the long‐lasting antitumor activity and indicate that this therapeutic regimen can help patients with advanced ESCC or GC to extend their progression‐free survival and overall survival.

This clinical trial has several limitations. First, the antitumor efficacy of each individual treatment component could not be accessed in this single‐arm clinical trial. Second, the sample size of this study was too small (15 ESCC and 15 G/GEJ adenocarcinoma patients) and no new safety signals were reported, therefore, the conclusions on safety and the data of survival are limited. Third, the results cannot be directly applied to Western populations because this study only enrolled Chinese patients.

Despite the limitations mentioned above, this phase II clinical study conducted by Xu *et al*. has evaluated the safety and preliminary antitumor activity of tislelizumab in combination with chemotherapy for first‐line treatment of advanced ESCC and G/GEJ adenocarcinoma. The results of this study lay a foundation for the application of tislelizumab plus chemotherapy in the first‐line treatment of ESCC and GC, although by analyzing the current data we cannot know whether the safety and tolerability profile of tislelizumab have been further improved when compared with other PD‐1 inhibitors. This is a new area and further research is needed. In addition, the efficacy of tislelizumab plus chemotherapy should be further confirmed by phase III studies with a global population.

## Disclosure

The author declares no competing interests.
